# IgG Antibodies against Measles, Rubella, and Varicella Zoster Virus Predict Conversion to Multiple Sclerosis in Clinically Isolated Syndrome

**DOI:** 10.1371/journal.pone.0007638

**Published:** 2009-11-05

**Authors:** Johannes Brettschneider, Hayrettin Tumani, Ulrike Kiechle, Rainer Muche, Gayle Richards, Vera Lehmensiek, Albert C. Ludolph, Markus Otto

**Affiliations:** 1 Department of Neurology, University of Ulm, Ulm, Germany; 2 Institute for Biometry, University of Ulm, Ulm, Germany; 3 Genzyme Virotech, Rüsselsheim, Germany; Julius-Maximilians-Universität Würzburg, Germany

## Abstract

**Background:**

Multiple sclerosis (MS) is characterized by a polyspecific B-cell response to neurotropic viruses such as measles, rubella and varicella zoster, with the corresponding antibodies measurable in CSF as the so-called “MRZ reaction” (MRZR). We aimed to evaluate the relevance of MRZR to predict conversion of patients with clinically isolated syndrome (CIS) to MS, and to compare it to oligoclonal bands (OCB) and MRI.

**Methodology/Principal Findings:**

MRZR was determined in a prospective study over 2 years including 40 patients that remained CIS over follow-up (CIS-CIS) and 49 patients that developed MS (CIS-RRMS) using ELISA. Using logistic regression, a score (MRZS) balancing the predictive value of the antibody indices included in MRZR was defined (9 points measles, 8 points rubella, 1 point varicella zoster, cutpoint: sum of scores greater 10).

MRZR and MRZS were significantly more frequent in CIS-RRMS as compared to CIS-CIS (p = 0.04 and p = 0.02). MRZS showed the best positive predictive value (PPV) of all parameters investigated (79%, 95%-CI: 54–94%), which could be further increased by combination with MRI (91%, 95%-CI: 59–99%).

**Conclusions/Significance:**

Our data indicate the relevance of MRZR to predict conversion to MS. It furthermore shows the importance of weighting the different antibody indices included in MRZR and suggest that patients with positive MRZR are candidates for an early begin of immunomodulatory therapy.

## Introduction

In over 80% of patients who later develop multiple sclerosis (MS), the disease initially presents with an episode of neurological symptoms due to a single demyelinating lesion known as clinically isolated syndrome (CIS) [1]. Given the importance of an early treatment of MS, the clinical challenge in patients with CIS is to identify patients at risk of future events that would confirm the diagnosis of definite MS [2], [3]. Consequently, there is an ongoing search for biomarkers that could help to evaluate the prognosis in CIS. Several magnetic resonance imaging (MRI) criteria as well as biochemical markers have been investigated as possible predictors of conversion from CIS to definite MS [4]–[9]. Besides history and clinical findings, magnetic resonance imaging (MRI) has become the most important tool to establish the diagnosis of MS. However, as shown in a meta-analysis evaluating the use of MRI in the diagnosis of MS, MRI studies tend to produce higher estimates of sensitivity and lower estimates of specificity particularly in short-term studies [10]. Cerebrospinal fluid (CSF) is a promising source of biochemical markers in MS, since the CSF compartment is in close anatomical contact with the brain interstitial fluid, where biochemical changes related to the disease are reflected [11], [12]. It has recently been demonstrated that oligoclonal bands (OCB) are an independent risk factor in CIS implementing an almost two-fold increased risk of having a second attack in all patients independent of MRI [9]. CSF of patients with MS is characterized by a polyspecific, intrathecal B-cell response with prominent antibody production against neurotropic viruses such as measles, rubella and varicella zoster, the so-called “MRZ reaction” (MRZR), which was shown to be detectable in CSF of 80–100% of patients with MS [13]–[20]. As shown in a small study on optic neuritis, MRZR appears to be more specific than detection of OCB for conversion to MS [19]. In the present study, we used an assay optimized for CSF measurement in a highly standardized manner to determine the prevalence and prognostic relevance of MRZR in CIS regarding conversion to MS in comparison to other markers like OCB and MRI lesion load [4].

## Methods

### Patients

In a prospective study of the Department of Neurology, University of Ulm (Germany), we collected CSF samples from patients with CIS that remained CIS (CIS-CIS) over a follow-up of 2 years and from patients with CIS that developed definite MS of the relapsing-remitting subtype (CIS-RRMS) over the same period [2] (Table 1). Informed consent was obtained from all patients, and the study was approved by the local ethics committee.

**Table 1:** Demographic data, CSF and MRI findings in patients with clinically isolated syndrome (CIS).

		CIS all	CIS-CIS	CIS-RRMS	S*
**n (female/male)**		89 (56/33)	40 (22/18)	49 (34/15)	NS
**Age [years]**	Median (Range)	38.5 (13.1–70.9)	38.4 (16.8–70.9)	39.4 (13.1–63.6)	NS
**EDSS**	Median (Range)	2 (0–5)	2 (0–5)	3 (0–5)	NS
**Measles AI≥1.5**	n (%)	37 (42)	11 (33)	26 (59)	NS
**Rubella AI ≥1.5**	n (%)	32 (36)	9 (24)	23 (49)	p = 0.03
**Zoster AI ≥1.5**	n (%)	32 (36)	12 (32)	20 (42)	NS
**MRZR**	n (%)	33 (37)	10 (25)	23 (47)	p = 0.04
**MRZS**	n (%)	19 (21)	4 (10)	15 (31)	p = 0.018
**OCB**	n (%)	74 (83)	27 (68)	47 (96)	p = 0.001
**MRI**	n (%)	59 (66)	21 (53)	38 (78)	p = 0.02
**Barkhof criteria**	n (%)	25 (28)	8 (20)	17 (35)	p = 0.125

CIS all  =  all patients with CIS, CIS-CIS  =  patients with CIS that remained CIS over follow-up, CIS-RRMS  =  CIS patients with conversion to MS over follow-up, EDSS  =  Kurtzke Expanded Disability Status Scale, AI  =  antibody index, MRZR  =  AI for measles, rubella, zoster, two or more AI ≥1.5, MRZS  =  MRZ score >10, OCB  =  oligoclonal bands in cerebrospinal fluid, MRI  =  two or more lesions in T2-weighted magnetic resonance imaging of the brain. NS  =  not significant, S  =  statistical significance. * CIS-CIS vs. CIS-RRMS

### CSF Basic Analysis and Determination of MRZR

CSF leukocyte count (cells/cu.mm), total protein (g/L), lactate (mmol/L), the albumin CSF/serum concentration ratio (Qalb), immunoglobulin G, A and M and OCB were obtained as previously described [29], [30], [31].

Antibody levels against measles (M), rubella (R) and zoster (Z) were determined using an ELISA according to the instructions as supplied by the manufacturer (Genzyme Virotech, Rüsselsheim, Germany; CE certificate). Of note is that CSF and serum were diluted into a similar protein concentration range allowing a measurement in a similar region of the standard curve. Quantitative expressions of the intrathecal immune response were based on calculation of the CSF/serum quotients (Q) of specific antiviral IgG antibodies (IgG[spec]) and total IgG (IgGtotal): QIgG[spec]  =  IgGspec[CSF]/IgGspec[serum], and QIgG[total]  =  IgGtotal[CSF]/IgGtotal[serum]). The intrathecal synthesis of antibodies to M, R and Z was detected by calculation of the corresponding antibody indices (AI): AI  =  QIgG[spec]/QIgG[total]. In case of an overall intrathecal synthesis of immunoglobulin above the reference range (Qlim), the Qlim was used instead of the QIgG[total]: AI  =  QIgG[spec]/Qlim, if QIgG[total] > Qlim [32]. The upper reference range of QIgG[total], Qlim, was calculated according to Reiber's formula [32]. AI values ≥1.5 were considered to be indicative of intrathecal IgG synthesis against the respective pathogen [17], [18], [32]. MRZR was considered positive if two or more AI values were ≥1.5.

### MRI Analysis

MRI scans of the brain and spinal cord were performed on a 1.5 tesla whole-body MRI (Symphony Siemens, Erlangen, Germany) according to a previously fixed protocol including T1-weighted spin-echo (SE) axial slices with and without application of gadolinium-DTPA as well as T2-weighted SE axial slices. Hyperintense lesion on T2-weighted MRI were analyzed for lesions >3 mm^2^ and quantified on hardcopies. For this study, the presence of ≥2 lesions suggestive of MS in T2 weighted MRI (MRI ≥2 lesions) as well as Barkhof criteria [4] were applied as diagnostic criteria. Spinal lesions were accepted as infratentorial lesions in our application of MRI criteria.

### Statistical Analysis

Absolute and relative frequencies were given for discrete variables, median and range for continuous variables. Differences between CIS-CIS and CIS-RRMS were analyzed by Chi-Square test and Mann-Whitney U-Test respectively on an univariate basis in an exploratory sense. P-values below 0.05 were considered to be significant.

A score of the effects of measles, rubella and varicella zoster antibodies (MRZS) was defined by logistic regression analysis with weights for the single antibodies calculated by using the regression coefficients [33]. A cutoff value for the score to achieve the diagnostic characteristics was chosen on the basis of a ROC-analysis.

Combinations of the variables will be examined for their diagnostic value. Sensitivity was calculated as (true-positive/[true-positive + false-negative]), specificity was calculated as (true-negative/[true-negative + false-positive]). The positive predictive value (PPV) was calculated as (true-positive/[true-positive + false-positive]), and the negative predictive value (NPV) as (true-negative/[true-negative + false-negative]). For all diagnostic values the exact 95% confidence intervals were given [34].

## Results

### Demographic Data Analysis

**Figure 1:**
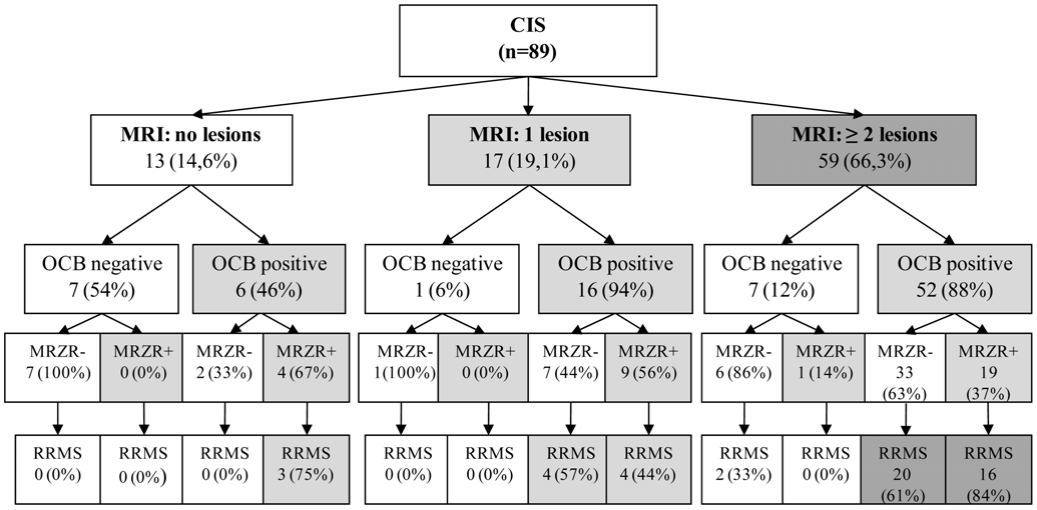
MRZR in patients with CIS. Figure shows biomarkers that help to evaluate the risk of multiple sclerosis in patients with clinically isolated syndrome (CIS). MRI  =  lesion load in T2-weighted magnetic resonance imaging (lesions >3 mm^2^), OCB  =  oligoclonal bands in cerebrospinal fluid (CSF), MRZR  =  MRZ reaction, antibodies to measles, rubella and varicella zoster virus in CSF with two or more AI (antibody indices) ≥1.5. RRMS  =  relapsing-remitting multiple sclerosis.

In total 89 patients with CIS were investigated (Table 1). Fourty-nine of these patients developed an MS within a time span of two years. The other 40 did not develop MS according to McDonald criteria (Figure 1) [2]. There was no significant difference in age, gender and EDSS distribution of these groups.

### Frequency of MRZ Antibodies in CIS

The conversion rate from CIS to definite MS over follow-up of 2 years was 55% (49/89). A positive MRZR was significantly more frequent in CIS-RRMS as compared to CIS-CIS (p = 0.04), as was a positive AI for rubella (p = 0.03), OCB in CSF (p = 0.001) and MRI ≥2 lesions (p = 0.02) (Table 1). Intrathecal synthesis of IgG antibodies (AI ≥1.5) against measles virus was found in 42% of CIS patients, followed by antibody synthesis against rubella virus (36%) and varicella zoster virus (36%). MRZR was observed to be positive in 37% of all patients with CIS (Table 1). It was positive in 25% of CIS-CIS and in 47% of CIS-RRMS (Table 1). OCB were detected in 68% of CIS-CIS and in 96% of CIS-RRMS. Barkhof criteria were fulfilled in 28% of all patients with CIS and in 35% of CIS-RRMS, while two or more T2 hyperintense lesions in MRI were observed in 53% of CIS-CIS and in 78% of CIS-RRMS (Table 1).

### MRZS

A score of the effects of measles (M), rubella (R) and varicella zoster (Z) antibodies was defined based on logistic regression analysis. Weights for the AI included in MRZR were calculated using the regression coefficients of M (β = 0.8922, p = 0.059), R (β = 0.8472, p = 0.090) and Z (β = 0.1127, p = 0.821) and rounded to 9 points for M, 8 points for R and 1 point for Z. This resulted in a MRZ-score (MRZS) between 0 and 18 points with possible results of 0, 1, 8, 9, 10, 17, 18 points. In the analysis the cutpoint of more than 10 points for MRZS was used to achieve the best PPV. A positive MRZS >10 points was significantly more frequent in CIS-RRMS as compared to CIS-CIS (p = 0.018, Table 1).

### Predictive Value of MRZR and MRZS in CIS

Of all markers investigated, OCB showed the highest sensitivity for conversion of CIS to MS (96%), which could not be improved by adding any of the other parameters (Table 2). MRZS (90%) and Barkhof criteria (80%) [4] showed the highest specificity of all single parameters. Barkhof criteria showed a higher specificity and PPV than MRI ≥2 lesions. MRZS showed the best PPV (79%) for predicting conversion from CIS to MS of all single parameters and was superior to MRZR (70%) and Barkhof criteria (68%). PPV could be increased to 91% by combination of MRZS with MRI (≥2 lesions) or by combination of MRZS with MRI (≥2 lesions) and OCB (Table 2).

**Table 2:** Sensitivity, specificity, positive (PPV) and negative (NPV) predictive value in percent (exact 95% confidence interval in brackets) for CSF and MRI parameters regarding conversion of clinically isolated syndrome to definite multiple sclerosis.

	Sensitivity	Specificity	PPV	NPV
**OCB**	96 (86.0–99.5)	33 (18.6–49.1)	64 (51.5–74.4)	87 (59.5–98.3)
**MRI**	78 (63.4–88.2)	48 (31.5–63.9)	64 (50.9–76.5)	63 (43.9–80.0)
**Barkhof criteria**	35 (21.7–49.6)	80 (64.4–91.0)	68 (46.5–85.1)	50 (37.2–62.8)
**MRZR**	47 (32.5–61.7)	75 (58.8–87.3)	70 (51.3–84.4)	54 (39.7–67.0)
**MRZS**	31 (18.3–45.4)	90 (76.3–97.2)	79 (54.4–94.0)	51 (39.2–63.6)
**OCB+MRI**	73 (58.9–85.1)	60 (43.3–75.1)	69 (54.9–81.3)	65 (47.5–79.8)
**OCB+Barkhof**	33 (20.0–47.5)	83 (67.2–92.7)	70 (47.1–86.8)	50 (37.4–62.6)
**OCB+MRZR**	47 (32.5–61.7)	78 (61.6–89.2)	72 (53.3–86.3)	54 (40.7–67.6)
**OCB+MRZS**	31 (18.3–45.4)	90 (76.3–97.2)	79 (54.4–94.0)	51 (39.2–63.6)
**MRI+MRZR**	33 (20.0–47.5)	90 (76.3–97.2)	80 (56.3–94.3)	52 (39.8–64.4)
**Barkhof+MRZR**	14 (6.0–27.2)	95 (83.1–99.4)	78 (40.0–97.2)	48 (36.2–59.0)
**MRI+MRZS**	20 (10.2–34.3)	98 (86.8–99.9)	91 (58.7–99.8)	50 (38.5–61.5)
**Barkhof+MRZS**	10 (3.4–22.2)	98 (86.8–99.9)	83 (35.9–99.6)	47 (35.9–58.3)
**OCB+MRI+MRZR**	33 (20.0–47.5)	93 (79.6–98.4)	84 (60.4–96.6)	53 (40.6–64.9)
**OCB+Barkhof+MRZR**	14 (5.9–27.2)	98 (86.8–99.9)	88 (47.4–99.7)	48 (36.9–59.5)
**OCB+MRI+MRZS**	20 (10.2–34.3)	98 (86.8–99.9)	91 (58.7–99.8)	50 (38.5–61.5)
**OCB+Barkhof+MRZS**	10 (3.4–22.2)	98 (86.8–99.9)	83 (35.9–99.6)	47 (35.9–58.3)

MRZR  =  AI for measles, rubella, zoster, two or more AI ≥1.5, MRZS  =  MRZ score >10, OCB  =  oligoclonal bands in cerebrospinal fluid, MRI  =  two or more lesions in T2-weighted magnetic resonance imaging of the brain.

## Discussion

OCBs indicating a polyspecific IgG response persist in the CSF of MS patients for many years [21], and MRZR was shown to be positive in 80–100% of patients with MS [13], [14]. However this finding was rarely evaluated systematically because of lack of CSF approved assays, that would allow a routine application which is not restricted to special laboratories [17]. The mechanisms underlying this “errant” intrathecal B-cell response in MS so far remain a matter of speculation [22]. A simultaneous co-infection of MS patients with different neurotropic viruses seems unlikely, and PCR for measles, rubella and varicella zoster virus was shown to be negative in MRZR positive patients with MS [13]. Measles, rubella, and varicella zoster infections are very common during childhood and may affect the brain more frequently than clinically observed [15]. They could induce the recruitment of long-lived memory B-cells, which CSF analyses have shown to constitute the majority of B-cells in the CSF [23]. The differentiation of memory B-cells into antibody secreting cells was suggested to occur independently of the antigen in a “bystander reaction” promoted by T-cells in MS [24], [25]. Consequently, the polyspecific intrathecal IgG response in MS is likely to mirror an enhanced B-cell-promoting environment, and may also reflect the individual's history of previous infections or immunizations [22]. This assumption is supported by the finding that in MS patients from Cuba a lower fraction of rubella-AI is observed, which may reflect a different immunization scheme in childhood [26].

Our data showed MRZR to be present in nearly half of CIS patients that later developed MS (Table 1, Figure 1). This finding supports the notion that immunological changes related to B-cell activation and intrathecal polyspecific IgG synthesis occur early on in the development of MS [22]. MRZR was significantly more frequent in CIS that developed MS over follow-up, indicating a prognostic relevance of MRZR in CIS. The occurrence of OCB in our patients was comparable to data in literature [9], [13], [15], [27], [28]. As expected, the sensitivity of MRI (≥2 T2-hyperintense lesions) was higher than observed for Barkhof criteria [4], though it showed a lower specificity for conversion to MS. In counting spinal MRI lesions as infratentorial lesions we went beyond the original brain-based Barkhof criteria [4], [33], but are in line with the current application of MRI criteria for diagnostics of MS [2].

The most relevant statistical parameters to predict conversion from CIS to MS are PPV and NPV, as the patient's disease status (CIS-CIS or CIS-RRMS) is unknown and the clinician needs to determine whether a positive/negative test result (for example the presence/absence of OCB in CSF) indicates that the patient really has/does not have MS. Our data showed MRZR to have the best PPV of all single parameters investigated and to be superior to Barkhof criteria [4], OCB and MRI (≥2 lesions in T2-weighted MRI).

So far studies investigating the relevance of MRZR in inflammatory diseases of the CNS did not weight the single AI included in MRZR [15], [18], [19]. Using logistic regression we found the prognostic relevance of varicella zoster to be much lower than the relevance of measles or rubella antibodies, which was found to be comparable. Consequently, we proceeded to define a score that would allow for the different prognostic relevance of the three AI included in MRZR. The PPV of the newly defined MRZS balancing the AI included in MRZ according to their predictive value was superior to MRZR and to all other single parameters (Table 2). Consequently, our data indicate the importance of weighting the different AIs included in MRZR. Our data suggest that maximum PPV can be achieved by combining MRZS with MRI (≥2 lesions), which showed the same PPV as combination of MRZS with MRI and OCB (Table 2).

Taken together, this study underlines the prognostic relevance of MRZR and MRZS to predict conversion to MS in patients with CIS. Our data indicate that CIS patients that have two or more T2-hyperintense lesions in MRI and have a positive MRZS are at highest risk to develop MS and should therefore be candidates for an early begin of an immunomodulatory therapy. Further studies are planned to confirm our findings and to evaluate a possible relevance of MRZR as a therapy response marker.
